# Supplementary material for: SOX9 and TCF transcription factors associate to mediate Wnt/β-catenin target gene activation in colorectal cancer
Source: J Biol Chem. 2022 Nov 22;299(1):102735. doi: 10.1016/j.jbc.2022.102735 (PMC9771724; doi:10.1016/j.jbc.2022.102735)
Supplement: Supplemental figures [file mmc1.pdf]

A

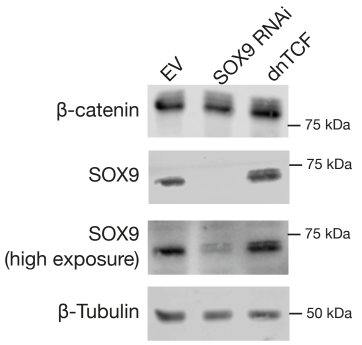

B

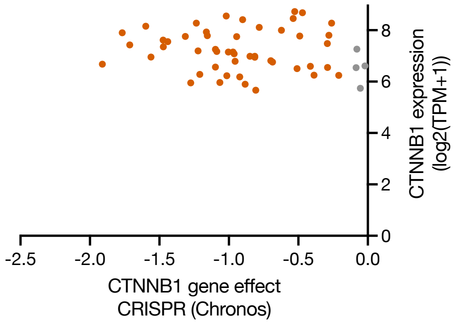

C

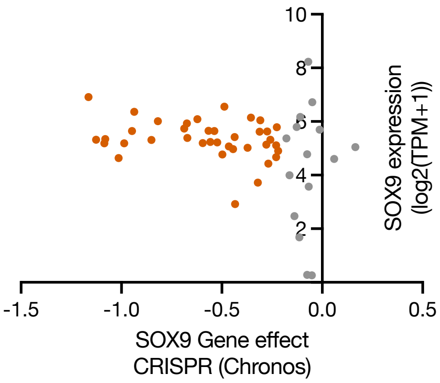

D

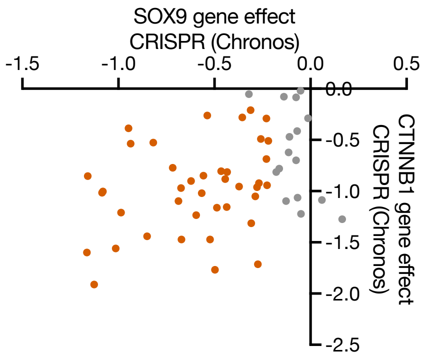

E

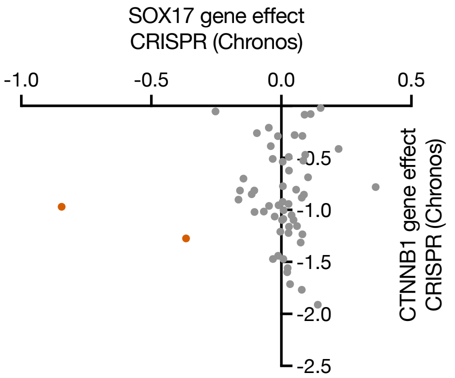

F

Gene	Entrez Id	Correlation
**SOX10**	6663	0.5211
**DUSP4**	1846	0.3923
**BRAF**	673	0.3753
**MAPK1**	5594	0.3561
**CYB5R4**	51167	0.3066
**MITF**	4286	0.2945
**CTNNB1**	1499	0.2928
**MAP2K1**	5604	0.2451
**FASN**	2194	0.2312
**NEDD9**	4739	0.2299

Fig S1. SOX9 is a regulator of CRC cell growth and proliferation.
A, western blots showing no change in β-catenin levels upon SOX9 RNAi and no change in SOX9 levels upon inhibition of Wnt signalling by dnTCF overexpression in DLD-1 cells. Cells were transfected with either the empty pcDNA3.1 vector (EV), or indicated plasmids and harvested for western blotting 48h later. *B***,** correlation between CTNNB1 expression and essentiality in 53 Cancer Cell Line Encyclopedia (CCLE) CRC lines. *C***,** correlation between SOX9 expression and essentiality in 53 CCLE CRC lines. *D,* correlation between essentiality scores of SOX9 and CTNNB1 (β-catenin) in 56 CCLE CRC lines. *E,* correlation between SOX17 gene effect and CTNNB1 gene effect in 56 CCEL CRC lines. In (B-E), essentiality (gene effect) is measured using Chronos scores. Lower scores indicate a stronger growth reduction upon CRISPR KO. Each data point represents a single cell line. Cell lines passing threshold Chronos score values of -0.2 and expression values of 2 TPM are coloured orange, while others are coloured grey. All data were sourced from the 22Q1 public DepMap release. *F,* top 10 genes with essentiality scores most correlated with SOX9 across all CCLE cancer cell line datasets. β-catenin shows the 7th best correlation.

A c-Myc-335 enhancer sequence: (Chr8:127400835–127401814)

CGCTCCATAGAGCCTGCAGAGGGCACTAGACTGGGAATTAGAAAACCTGATTTCCCTTCCAGCTCCACCTCTGACCAATTGCCTGACCC
TGGTCAAATTGCTTAACCTCTTCCTATCTCAGCTCCCTATCCATAAAACAGAGGGACGAATAAACTCTCCTCCTACCACTAAGAGGTGT
AGCCAGAGTTAATACCCTCATCGTCCTTTGAGCTCAGCAGATGAAAGGCACTGAGAAAAGTACAAAGAATTTTTATGTGCTATTGACTT
TATTTTATTTTATGTGGGGGAGGGAGCCGGCCCCAGCTGGAAAGCTGCTTTCTCTGAATCAAAGGGCAGGAACCCAGCAAGTTTCTCAG
GATTGGGGCCTTAGACTGGGCTGTGTATACAGACAGTGCCAGCCAACCCCACAGTTCAGTTTCCTTTAACCTGGTGCTCCAGGCAATAA
CTGTGCAACTCTGCAATTTAACAATGTGTTCTTTGTCCCACAACTGTTCTCGTTTCTCAACTGCCCAGGTAATATGTTTGGGCCTGTAG
GAAGAGTCAAATAGTTAATAAGGGAAGGGTTTGGCATGCCCTACGTAAGTTCTACCAGCAAGTCCCAACAAGAAGGCATTCTGTGTCTC
CTGATTCCTGACCTACCCCCAAAATGTACAAATGTACAAGGAATGAGCCCACTTTCCCAGCAGGCTGTAATACCAGTTTGGCCTATATC
AATGCATTGGTGAGCTGTGTTTTGTTTATGGTTTTATGCCATCTATTTTCCCATGGATATTATGTTTTCTAAAGAGCCCTTAAGTTTAC
GTCAGCTTTTAAAGCTACCAGCAGCACCATTTCAGTTCATATTAAGCCCTTAATATGGTATGAATAGGAGAGCTATTAGACTAAAGAGC
CATAATCATCCCTGAGGAAAACATCCATCACCAACATTTATGTGGTCCCTGAACTTCTAAAAGGTGTCATCTCTCTGGGGTGTATCTGG
T

Legend: **TCF sites** CDX sites CAG sites **SOX sites**

B

Probe	Sequence
WT	CAATTTAACAATGTGTTCTTTGTCCCACAA
5' mut	CAATTTAA**ACC**TGTGTTCTTTGTCCCACAA
3' mut	CAATTTAACAATGTGTTCT**GGT**TCCCACAA
5'+3' mut	CAATTTAA**ACC**TGTGTTCT**GGT**TCCCACAA

C

Mouse homologue of c-Myc-335 (chr15:61490400-61491920)

CATGGCAGAGGTCTTGAAAGCATGTATAAACAAACACACACACAAAGATCACAAACAAAATTCTAGACCCTATTCTGTAGCTAACTACA
TGTTTGACTTTGGAAATTGCAAACATTTTCCCTATCTTCATTGTTCTCTACTGACATCAACAAAATTACTTCAAATTTCTTTGTCAGTT
CATTTAGCCAGGACTCCAGCAAATGTATCAATCCATTTATCCTGCAGGGGGAGCTAGACCGGGTTCTGAAAACCCCGGTTTCTCTTCCC
AGCCCCATACTGGGTGATTGTGTGACCCTGGGCAAACTGTTTAACCTCCCCATATCTTTAGGTATCGTCCATAAAACAGAGGGATGAAT
AAACTCTCCTCCTACCACTAAGGGGTGTAGCCAGGGTTAATACCCTCACCATCCTTTGAGCTCCGCAGATGAAAGGCACTGAGAAAAGT
ACAAAGTATTTTTATGTGCTATTGACTTTATTTTATTTTATGTGGGGGAGGAAGTTTGCCCCAGCTGGAAAGTTGCTTTCTCTGAATCA
AAGGGCAGGAACCCAGCAAGTTTCTCAGGATCAGGACCTTAGGCTGGGCTGTGTGTACAGAGTGCCAACCAACCCCACAGTTCAGTTTC
CTTTAACCTGGTGCTCCAGGCAATAACTGTGCAACTCTGCAATTTAACAATGTGTTCGTTGTCCCACAACTGTTCTTGTTTCGCAATTG
CCCAGGTAATATGTTTGGGCCTGTAGGAAGAGTCAAATAGTTAATGAGGGAAGGGCTTGGCATGCCCTACATAGGTTCTTCCAGCAAGT
CTCAGCACGAATACATTCTCTTGCTCCTCTCTCCTCTGCTTCTTGACCTACCCTCCAAATCTTGCCAGTGTCACAACCAGGAATGAGTC
CTCTTTCCCGGCAGGCTGTCATACCAGTTTGGCTGTTATCAATGCCTGGGTGAGGTGTATTTTGGTTATAGTTTTACACCATTTATTTT
CCCAGGGATATTATGATTTCTAAGGGCCCTTGGGTTTGCATTGGCTTTTCACACTACCAGCAACATCATTTCGATGCTTATTAAGCCTT
TAGTATGGTATGAATAGAAGAGCTCTTGGACTAAAGTACCATAATCATCCCTAAGAAAAGCGTCCATCCCCAACCTTGACGTGGTCCCT
GAACCTCTAAGAAGTGTCATCCCTCTGAGGTTTGTCTGATGAACGCTTTCTCTGGGAGATCCCAAGAAGTCACTGTATCAGACACAGCT
TAGAAGGGCACTTTCTAGCCATTACAAGTCCCCTAGTTGAGCATCTCCATTTTGTCTACGTGATGAGCCTTAATTAAATTTGCCAGGGT
TTGATGCTTTACTCCCAGATGTGGGGCTGACAGAGATTGCTGACATAAATAAAGTGTGCTCCAAGTGTTTGCTCAGCTCTGGGCTCAGC
CAGGACAGATGCAGCTTCTTGAATGTCATTCCCAAGATATCCTGGAGGTATTTAGCTTCCCTCCCTCTACTCTGGAAAAGATACCATGG
GCAACATC

Rat homologue of c-Myc-335 (chr7:93157287-93158520)

TGACCCTGGACAAACTGTCTAACCTCCCCATATCTTTAGGTCTCATCCATAAAACAGAGGGATGAACAAACTCTCCTCCTACCAGTAAG
GGGTGTAGCCAGGGTTAATACTCTCACCCTCCTTTGAGCTCAGCAGATGAAAGGCACTGAGAAAAGTACAAAGTATTTTTATGTGCTAT
TGACTTTATTTTATTTTATGTGGGGGAGGGAGTTTGCCCCAGCTGGAAAGTTGCTTTCTCTGAATCAAAGGGCAGGAACCCAGCAAGTT
TCTCAGGATCAGGACCTTAGGCTGGGCTGTGTGTACAGAATGCCAACCAACCCCAGAGTTCAGTTTCCTTTAACCTGGTGCTCCAGGCA
ATAACTGTGCAACTCTGCAATTTAACAATGTGTTCGTTGTCCCACAACTGTTCTCATTTCGCAATTGCCCAGGTAATATGTTTGGGCCT
GTAGGAAGAGTCAAATAGTTAATGAGGGAAGGGCTTGGCATGCCCTACATAGGTTCTTCCAGCAAGTCTCAGCAAGAACACATTCTCTT
TTCTCCTTCTCTCCCCTGGTTCCTGACCTACCCTCCAAAACTTGCAAGTGTCACAACCAGGAATGAGTCCTCTTTCCCGGCAGGCTGTC
ATACCAGTTTGGCTGTTATCAATGCCTAGGTGAGGTATATTTTGTTTATAGTTTTATGCCATTTACTTTCCCAGGGATATTATGATTTC
TAAGAGACCTTGGGTTTGTATTGGCTTTTCAAGCTACCAGCAATGTCATTTCAATGTTTATTAAGCCCTTAATATGGTATGAATAGGAG
AGCTATTGGACTAAACTACCATAATCACCCCCAAGGAAAACATCCATCACCAACCTTGATGTGGTCCATGGACCTCTAAGAGGTGACAT
CTCTCTGAGGTGTGTCTGATGAGAGCTTTCTCTGGGAGATGCCAAGAAGTCAGTGCATTTGACACAACTCAGAAGGGCACTTTCTAGCC
ATTACAAGTCCCCTAGTCGAGCATCTCCATTTCATCTACGTGATGAGCCTTGATTAAATTTGCCAGGGTTTGATTCTTTACTCCCAGAT
GGGGGGCTGACAGAGATTGCTGACATAAATAAAGTGTGCCCCGAGTGTTTGCTCAGCTCTGGGCTCAGCCAAGACAGATGTGGCTTCTT
GAATGTCATTCCCAAGATATCCTGGAGGTATTTAGCTTCCCTCCCTCTATTCTGGAAAAGATACCATGGGCAACATC

Fig S2. The c-Myc-335 WRE is bound and regulated by SOX9.
A, sequence of the c-Myc-335 WRE with regulatory motifs annotated. Positions shown are based on the GRCh38.p13 primary assembly of the *Homo sapiens* genome. *B,* sequences (5'-3') of the SOX site probes used for EMSA. The SOX sites in the WT and corresponding locations in the mutant probes have been underlined. Mutated bases are emboldened. *C,* sequences of the mouse and rat homologues of the c-Myc-335 WRE with TCF and SOX sites annotated. Positions are based on the GRCm39 (mouse) and mRatBN7.2 (rat) genome assemblies. TCF and SOX sites conserved in at least 2 organisms are underlined.

Human Myc+8 enhancer sequence:
Chr8:127742878-127744069

GGTCTTCAGCTCCCTGTACTTTGGGATTTTAATCTACCACCACCCATAAATCAATAAATAATTACTTTCTTTGACTCTGACTCCTAGAA
TAATCTATTCAAAACCTTAATGTCTTTTTCTTGATCCTTCTTTTGAGTCCTAAGTACaCATTACAGCTTCAAATTGGCACGTCATATAG
GCGAA**TTTCAAAGG**G**AGATGCAA**TCCACAGAAGTATAGTA**GTTCAAAGG**GTTACAAA**AGCAAGGC**G**CTCTTAAA**CAGCTCAGTCTTTGC
C**CCTTTGTGG**CCTAGGGCTGGAGTGCAGCTCTGGGGTGACTCACTTGGGAATCGGGAAGGTGTTAGTCTGAATCACTAAGTCCAGGCAA
GCCCTCAGAATAGGAGAGAGTGTTCCTAGCAAGGAAAACAACTCTCCATTCCAAATAATCAGGAAAGAACTTTAGGGATGTGGAGCTTG
GCTATGGGAATAGAAAGGAACCATTCCAAGTGCCTATTAGGCCGCTCTTACCTTTACTGAGCCAGAGAATGGCTCTGAAAACAGGACAG
ATGCCAACTTCCTTCCCGAAAGTCAGGCTGATCTTGACCACAATACAAATTGGCCCTTAGAGCCTATACAGGGAGTCCCAGGGGTCTCT
GCCATTGTGCAACCTATTTTGTAGATAATAATCAAGAATCGGACGTGAAGGGGAGGAGTTTGCAACTTGGTCAGGAATGTATAAGAAGG
AATAAGCTAATTCTGACTATGCCCTTTATCCATGACACTATCCAGGAATTAATGACTCTCCCAGAGGATTCCTGGAATGATTTTGTTGA
GGGATGGAATGTATAAAGAGGAAGGAAGTGTTATTTTATGCTGCCATTTGGAAG**CAACAAAGG**AGATCAACAGTATGAAAACAATCAAT
CAAATTTGAAAATGAACAAAGTTTTCACAATCCCAGCCTAATACTTAGAGAGCTCACAGCTTGGATGCATAAGTAAAGAGTTCTCTGCT
GGTCTTTAAGACAAACTCTCACACAAAACTTGGGAAAAAGGACAAAAATGTTGCATTAGGGGGTTTTCTGTGGTTTGTTTGCAATAACT
ATAATTGGCTCA**ATCAATAA**T**TATTTTTT**AGTATACACACTAAGGGCCCCTGTAGCATTTTTTCCCATCG**ATAAATAA**T**CCTTAGTC**TA
GAAAATGCCGAGGGATGTTCTCCACCCTTGTCT

Mouse
Chr15: 61863502-61864717

GGTCTCTAGCATCCTGGAGTGTCTTTGGGACTTTAATTCACCATCATTCATAAACCAATAAGTAATTACTATCTTTGACACCCCCCCCC
CATGATCTATTGAAGCGTTTAACATTTAG**TTTTTGATC**TTATTTTTGGCTCTTTTAGACTCACCCTCAGCTTCAAACTGTTACACCCTC
TATCCTACA**TTCAAAAGA**AAACACAAACCCCAGTAGTAGTTTGAAAT**ACTTTGAGT**A**GTTCAAAGG**ATTAGCAAAAGGGGGGAGCAGGG
GGTG**CTCTTAAA**TAGTTCCCTCTTTTTCC**CCTTTGTGA**GCCTAGGCTGGAGTGCAGCCCTGGGGTGACTCACTTGAGACCTGGGAAGGT
GTTAGGTTGAATCACTCAGTCCAGGCAAGCCCAAAGAATAGAGGAAAGCATTCCTCATTAGGAAAACAACTCCTGTTCCAAATGATCAG
GAAACAAGTTTAGAGATTCAGATTTGGCTGTGGGGATGGAATCGAAGTATCACAGCTCCCTATCTGGGCACTTCTCAGCTTTACCAAGC
CAGGGAATGGTCTGAAAACAGGACATCGGCCAGCTTCCTTCCAGAAAGTCAGGCTGATCTTGACCATAACACAGGAAGGCTCTCCCAGC
CAATCTGGAGTCCCAGGGGTCTGCAACATTGTGCAATCAAA**TTTATAGA**T**AACAATCA**CGATGAGGGGTGAAGTGGGAGGAGCTTTCAG
CTTGGTCTGGGATATACAAGAATTAGCTAGTCTCTTCTGTTGTTCACCCATGACACTGGAACTCAGTTTCCCCGAGAGGATCCCTGGG
ATGGTGCTCTTCAGGATAAACTGAGTGAGGAGGAAGTGTGACTTTATGCTATCATTCGGGGACAACACTAAAAAGCAATCAATTACACT
TTAAGTTGAACAAAAGTTTCACAATACCAGGATAGTGCTTGAGTCATCCAAAGCTTGAACACGTATGTAGAGTCTTCTGCTGTTCTTTA
AGATGGACTTTGGCACTAACCTTGGAAAAGAGGGCCAAAAAACCCACCCCCCAAAAACAAACAGAACAAAAAACGTGAGGGGATTTTCT
GT**GGTTTGTTTACAATAA**GAGATGAGTCACAATAATGATTTTTTTTTTTTTTTTAGCATGACTGCTAAGGAACCCTGAAGCATTTCTT
CCCAACAAAAACTAATCTTTAGGTTATAACATGCTGGAAGATGAGCTTCAAGCCTGTCTA

Rat
Chr7:93599915-93601095

GGTCTCTAACATCCTGGACTGTCTTTGGGATTTCACCATCATTCATAAACCAATAAGTAATTACTG**TCTTTGAT**ACACACACCCCCTAA
GATAATCTCCTGAACCATTTAACACTTAG**ATTTTGATC**TTTTTGGCTCCTTTAGACCCACACTCAGCTTCAAACTATCCCACCCTCTAT
CCTAA**ATTCAAAGG**AAAAGACAAACCCCAAACACTGAGTA**ATTCAAAGG**GTAACAAAAGGGGGCGGGGGGGCGGGGGTGGTGTGCTCGT
AATAAGTTCCCCCACTTTGC**CCTTTGGTG**AGCCTAGGCTGGAGTGGTGCCCTAGGGTGACTCACTTGAGACCCGGGAAGGTGTTAGATT
GAATCACTCAGTCCAGGCAAGCCCAAGGACTAGGAGAGCATTCCTCATTAGGAAAACAACTTGTTCCAAATGATCAGGAAACAAGTTTA
AGGATTCAGAGTTTGGCTGTGGGAATGGAAGGGAAGTATCACAGTCCCCTGTCAGGGCACTTCTCAGCTTTACCAAGCCAAGGAATGGT
CTGAAAAACAGGACATCAGCCAGCTTCCTTCCAGAAAGTCAGTCTGAGCTTGAACATAACACAGGAAGGCTCTTGGTAGCCAATCTGGA
GTCCCAGGGTTATGGGACACTGTGTAGTCAAA**TTTATAGA**T**AACAATCA**CAATGAGGGGTGAAGTGGGAGGA**GCTTTCAAC**TTGGTCTG
GAATATACAAGCATTGCCTGGTCTCTGTCGTTCACCCATGACGGTGGAATTGAAAGCTGCCCCAAAGGATTCCTGGGATGGTGTTCTC
CAGGGTAGAACAAGTGATGAAGAAGTGTTATTTTATGCTGACATTAGGTGGCAACATGGGAGACCAACCTTACGAAGCAATCAATCAAA
CTTTAAGATGAACAAAATTTTCACAATAACAGGAGAGTACTTGGACCATTCAAAGTTTGGACGTGTACAAAGAGTCTTCTGCTGTTCTT
TGAGACAAACTTTGGCACTATGTTTGGGGAAGAGGACAAAAAGTAAAATGTGAGGGGATTCTCTGC**GGTTTGTTTACAATAA**GCGATGA
GTCACAATGATGACTTGTTAGTATCGACTGCTTAAGGGACCCTGAAGCATTTCTTCCCAACAAAAAGTAATCATTAGGTTATAACAAGC
TGGAAGACAGGCTTCAAGACTGTTT

Legend: **TCF sites** **SOX sites**

Fig S3. Sequences of the human Myc+8 WRE and homologues in mice and rats.
Positions shown are based on the GRCh38.p13 (human), GRCm39 (mouse) and mRatBN7.2 (rat) genome assemblies. Putative TCF and SOX9 binding sites are annotated and sites conserved in at least 2 species are underlined.

Human Myc-29 enhancer sequence:
Chr8:127706818-127707901

AAAAGGTTGGGCTGGTTTCAAATGTTCTCAG**AACCAAAGC**AACACACGCCTTTCAGAGCGCTGGAGAAAATGCTGTTGAACAAGCTCTC
ATGGCTCAACAGTGGAGATCTGAACAATGTATCTGACTATTAACATAATTTGAGAAGAGAGGCTCCCTCTTAACCTGTATTTTTGGCTC
ACTCAAAAAAAGTGCTCCTGTCAGCCTTCAATTTCTCTACCTGGGTCTCTAGTCTGAGGACAGCATCCTTTTGGAAGACTTCAGCAATT
TCTGTGGAGCCATGTTTAAATTGGGCAAGTGGAAAACTAACTCCCTTTA**AGAAAAGT**G**GCATTGTT**TTGCAGTCAGGTAATTCCACGTG
GGCTCACTGGTTACTTTCCATGGGAATAG**TCTTTGAGG****AAGAAAGG**G**CCTTTGTTG**AGATTTGTTTTGTTTTGAGAACAGTCAATTGGT
TCTTTCTACTCTTCCTTCTAGGGCGCCAATCTTTCCAAGGATAA**CCACAAAGT**GCAAGCAAAAATAATGAAAAAGGAAAGAAAGAAAGA
AAATCTGTATGCAAACCCAGGTTGTCATTATCTGGCGTTGTTTTCGGCCATTCTTGTCTTCTGACTCCTACCTGGCACACAGATCACTC
CTGCCCACTCCTGCCCATTACTCTCTATCTTGTACACGCCTTGCTGGTACATATGGAGGACCATGGAAGAAAAGCATTGCTGTAAATTT
CCTCCTT**CTCAAGGA**A**TCCTGCTC**CCTGAGTTTCTGCTTAAGTGACTTCTCACCTTCAAACTCACTGAGGTTATTCTTGTTTGGCTTCA
GATAAGTGGCTGAGATTTTAATTGTTCTCAATTGGTCTGTTTATCAGGGTAACTGTTTCAGTTTTCTTCAAGACAAATGATTAGATCCT
TCGGTCTTTATCAGTATATTAAGTATGTTTTTCAGGTGTGGACTTAACTTTGCCCTCCACTCCCAGCAACCGTTTCCTTTCATAACTTG
GGTGTAGTTTAAACACACACACACACACACACACACACACACACACACTTGCTCAGAGTTATTTTGGAAAGTGTATTCTTTACCAGTGA
CACCAACCTCAAAAGA

Mouse
Chr15:61831213-61832304

CAGATACTAGGCCCGCTTAAAAAAAAAAATCACAG**AACCAAAGC**AACTACCCATTGCTGAAAGAGCCAAGAGAAGATATCAGACAAGGAC
TCTGCTCAACAGCCAATATCCAAACAGAGCATCTCACTGTGTGCATTGGCATAGTCCAAGCAGGGATAGGAAGCTGCTGATTTTTTTTT
TTCACTCAAAAGGAAAACATTCCTTTTAGCTTTAAATTTCTCTGCCTTGGTCTCTCATCTGAAGACCACATCCTCTTTGAGGACTTGGA
CAGTTTCTGTGGAGCCATGTTTAAATTGAGTGAGTGAAAAGCTAACTGTCTTTTT**ATAAAAGT**A**GCATTGTT**TTGCAGTCAGGTAATTTC
GGGCCGGTTCAGCTTTTACTTTCCATGGGAATAG**TCTTTGAGG**AAAAAAGGGG**CCTTTGTTG**AATTTTGTTTTGTTTTGAAACTAGTCA
GTTAGTTCTTTTTACCTTTCTTTTGGGGGGCGCCAGTCTTTCTAAAAACAACCACAAAATTCTGGAAGAAGTAATGAAAAGAGGAAGAA
GGAATCTGTACACAAATCCAATTTGTCGTTATCTGGGATTGATTTGTCTCCCTTGCCCCCAAACCTAATAAGTACATCGTGGGATCCTG
CCCAAAATTTCTCATTTCATACATCCCCAGTGTCTGTATGCAGAGGATCACTCGGAGGGCCATGGTAAAAATGTCCTCTTTCCCAGTGG
ACACAGGAAACTGCCCCCTGGGTTTCTTCTCAGGAGATTTCTCACCGGCAGACTTTCTGTTCCAATCCTTGCTTGGTTTCGGAAAAGTG
ACTGAGATCCAGAGTGCTCTCAGTAGGTCTGTTTATCATCACAAACTGCTTCAGCTTTCCTCAGGACACAGGGTAGGTAGTTTATCTGT
GTATTGGGGTGTGTTCTTCAGGTGTGGAGTTAACTTTGTCTTCCGCTTCCGGCTGCAGTTCCTCTTCCTTAATTAGATATACATTATTT
GAGCATGCGCACATGCTCGCTCTCGCACATACACAGAGCGAGAAACAGAGAGACAGAAACACAAAGGCAGAGAGATAGAGGCAGAGAGA
CAGAGACAAAGAGAGCCCAAAAGA

Rat
Chr7: 93553494- 93554583

CTGATACTAGGCCTGTTTGAAAAAATTTTTTTCACAG**AACCAAAGC**AGCTACCCATTGCTGGAAGAGTAGAGAGAAGATATCAGACAAG
AAGTCTGCTCAATGGCCAATATCTGAACAGGGCATCTCACTGTGTGCATTGGCATAGTCCAAGTAGGGATAGAGAGCTACTGATTTTTT
TTTTGGTTCCCTCTAAAGGAAAACATTCCTTTTAGCTTTAAATTTCTCTGCCTTGGTCTCTAGTCTGAAGACCACATCCTCTTTGAGGA
TCTGAACAGTTTCTATGGAGCCATGTTTAAATTGAGTGAGTGAACAGCTAACTCCCTTTT**ATAAAAGT**A**GCATTGTT**TTTGCAGCCAGG
TAATTTCAGGCGGGTTCAGCTTTTACTTTCCATGGGAATAG**TCTTTGAGG**AAGAAAAGGG**CCTTTGTTG**GATTTTGTTTTGTTTTGAGA
CTAGTCAGTTTTTTTTTCTATCTTTCTTTTGGGGGCGCCAGTCTTTCTGGAAAAAAAAAGAAGAAAAACAACTACAAAATTCTGGAAGA
AGTAATGAAAAGGGGAAGAAGGAATCTGAACACAAATCCAATTTGTCATTATCTGGAATTGATTTATCTACCCTGCCCCCAAACCATTG
TGGAATCCTGCCCAAGATTTCTTATTTCATACCTCTCCAGTGTCTGCGTGCAGAGGAGCACTGAAAGAGCTATGGTAAAACTGTCCTCT
TTCCCAGTGGACAACTGGAAACTCTGCTCCCTGGGTTTCTTCTCAGGTGACTTCTGACCTGCAGACTTTCTGTGCCAACTCTTGTTTGG
TTTCAGATAAGTGGCTGAGATTGTAATTGCTCTCGGTCGGTCTGTTTATCATCGCAAACTGTTTCAGCTTTCTTCAGGACATGGGGTAG
GTAGGTAGGTTCTCCAATGTTTATCTGTGTGATGGAGTGTGTTCTTCAGGTGTGGAGTTAACTTTGCCCTGAGCTTCCGGCAGCCGTTT
TCCTTCCTTAATTAGGGTGTACATTGTTTAAGCATGAGCAGAGAGACAAAGAGACAGAGAGACAGAGGCAGAGAGAGAGACAGAGAGAC
AGAGACAATGAAAACTGAGAGA

Legend: **TCF sites** **SOX sites**

Fig S4. Sequences of the human Myc-29 WRE and homologues in mice and rats.
Positions shown are based on the GRCh38.p13 (human), GRCm39 (mouse) and mRatBN7.2 (rat) genome assemblies. Putative TCF and SOX9 binding sites are annotated and sites conserved in at least 2 species are underlined.

Human Myc-521 enhancer sequence:
Chr8:127214627-127216810

```
ACAGTGTTAGACAGTACAAAGGGCATTTTTACCTCCAACACATCTTTCATGAAGAGTTTTTTATCTAGGTTTCATGGAGCCCTACGGTAAATAAATTTGCCCAAGTT
CTAACAAGATTCTGGAATCTCATATGTGGGTATGTTCATGTTGGGGTGTGTGTGTGTGTGTGTATGTGGGGTGTATGGGTGTCTGTGTTTTTCCTCTGAAAAGCACC
ATAGTCTCTGCCACATTTTTAAAAAGGTGCATAACCCTTTAAACAGGAACCATTTACTTTAAATGGAATCAGAAATCTCACTATAAAAAAGCAATAATATTAATTTC
TACTGTGTGTGTCTCTTGCCTTTTATTCCATGTTCACAATTCATCTTATTTTCATTTAAGACAACCAACTGGGCTAGTTAGAGCCGAGATTTGAGCCCAGTTTTTCT
CACTGACTCTAAAACCTATCCATGCTCCTAAACCTCCTCACCATTGGAGGGCATTGCTGTTTACCCTTTCAGTTTCAGCTGTACTATCAAAAGCAGCAAATCAAGGG
CAGGGACCACAGCTTGACCTTAAACTATTCAACTGCACTTAATTACCCATTGTAGTGGAAGCACATAAATACAGGATGTCCCCTCACTCTGCCATTCTGCTTTGCAT
CATTATCAAAAAGACAAAGGTAATTCATGTCTAATTACTCACTCACCGCAAGGTCAGAATATTTATTTTTATCATACTTGTGATTTTTAGGATTAGTGATATCAGTA
TTTATTCATGGCTAACCTAAATCAAACATCCTGTTGATATCTGTAAAGAGCCATATAGTTATTGTCGCAGGTATGCTGAGTCACAAAGCAAATACAAAGTTTATGAG
TTGACAGGGAAAAGGCTGCAGAGAGGTAGAGATAAGTGTGCCTGGGTAATCACAAGTGTCTTGTGAAATATACACATTGTTTATTTATATTGTCCATTTATCAAGCT
CAATGGGGGCTTGGGAAACTGAAAATAATCAAAATAGGAAAAATTATTGGTATTATTTTCTAAATTTGCTGCTGGTGCTGACTTTTTGGTTCAATTTATTAAATACT
TACTTTAGAAATTATTCTCTGTTTTTAGTGGAATAGAGCTTTGACCAGCTTGAGAAAGACAAAAGGAGAAGTAGAAGAGAAGGGGAAATAAATGAGGCTCATGTGAC
TGGGATTAATTTACTCAGAGATCTGGAGTAGCAATAACTTGTGTCTTTCCAAAACCCCCGCAGTCAATTAATGAGATCAATTTAAAGTGGCAGTATTGAAAGGGAAA
ACCCAAACACTTAGATATAGCAGTATGCAACTGACCTATTCCTTTTTCCATTCAGATTCAGTCAGTCAAATATATCTTATTGACTTTGACTACTGTCATCATAACTG
TGTTAGCTAATTGTCTGAAGTTTTCATTCATTCTCTGTCTTTGCAGATGTCACTGCTATTCAATCTGTATTTCCTTGCTCTGTCTCGCTGTCTA*TCTGCACTAACA
GGATATACACGTGTGTGTACACATTTTTTTTTCTTAGTCTATCCCATTGTTTCTCTTGATGTCCTTGGACAGATCAGCAGTCTGAAAATCAGGCTTCTAATAGAGAG
TCTTACTAGAAGTAACTAGCTGTGAAACTTGGGCAAGTATTTGACCTCTCCCTTCCCCCATCTATGAAAGGAAGATGTGAAAACAAATGGTTCTGTGGGTCCTCTCC
CACTTATTCACTCGATATTTCTATAATTTTCTATCTAGTTTCCATGGAGCACAAGGTCATTAGTAATGTGCCCCATGACTCTCAGATTAATCAGACAAGCCAACACT
GGGGGAGATGTGAACAAAGTCTTAGAGTCTAGTTGATAGTAAGAATATGAAATGAATATGACTCACTGATGTGATATAGTCTTCAAAAAACTAAATTTGGTGTTTGA
ATGCGTAGAAATATATTAGGTTTTACAACATAAAATTTCCATTTTGGTAGATGGAAAATGTTGAATAAAGGCAATTTCATAAGGTTCATAGTCTGTAGAGTGAAATG
GAAGTGGAAAAACCTCACTTTGCTCTGTTCTGCTCTACTTAGCTCATACTTGAAGTAATCATTCAGTTCTTGATGCCATACAGACAAAGAAGGTAGTGGACAAAAAG
AGACACACATATTCAGTAGATAGCAACCAAGCTGGGAGGGGAAC
```

Mouse
Chr15: 61317631- 61319835

```
AGCCATTTATTTATTTGTTTGTTTATTTATTTACACCAAAACACCTTGAATCAATAAATTGTGATCTAGATCTCATGCAGCCCTAAAAATCTGTTTGCTTAAGTTCT
AAGAACCTTCTGGAATTTCATAAAAACAGCATTGAATGTATGCACATAAATATGTTTAGGGCTGTATTCCTCCTCTGAAAAGAGATCTTCTAGCATTTATTGTAATG
TAATTCACCACTGTTCATAAGGGTTAAGAGCCATTGACTTGAAATGGGGCTAAAAACATAAATTATTTATAGAGAAGCAATAATATTAACTTATATATTACAAGTCT
CTTACTATTTATGACATGCCCATAATTCATCTCACTTGAGCTCAAGACAGGCAGCTAAACTAGGCATAGCATAGCCCAGAGTAACAACTAGCACTGTGCTCCTGGCC
CTTGCTATTGGAGGGCATTGGTGTTTACCACCCCCACTTCAACTGCACAAAGCAAGGGCAAGGACCACAGTATGACCTTAAACTATTCAACTCCACTTAATTACCCA
TTGTAGTAGGAGCACATAAATACAGGATGTCCCTTTGCTCTGCCATTCTGCTTTGCATCATTATCAGAAAGACAAATGTAATTCATGTCCAATTACTCACTCACCGC
AAGGTCAGAATATTTATTTTTATCATACTGGTGATTTTTTGGATTAGTGGTATCAGTATTTATTCATGGCTAACCCAAACCAAACATCCTGTTGATATCAATGAATT
GCTATATAGTTATTGTCACAGGTATGCTGAGTCACAGAGCAAATAAGAAGTTTATGAGTTGACAGAGAAAAGGCTACAAAGAGGTTAAGATAATGTTCCCTGTTAAT
CACAGGTGTCTTGTGAAATAGATGCATTGTTTATTTATATTGTCCATTTATTGAGCAGTCGGTGCTTGGGAAACCGAAACTAGCCCAAATAGTGAGGATTAAATAAA
ATTCACATCATTATCTTTAAAATTGGTTGCTGGTACATTTTGGTTCAATTTAAGAACAGCTCATTTTAGCAATCAGTTTTTATCCTCTGCCTCGTAGCGAAGAGCTT
TGTCTAGCTTTCAGATGTAGACAGAAGTAGGAGAGGTGGAAAATGAAAGAAAACTCTTTTGGGTATGGTTCATTTGCTCAGAGATCTGGAGTAGCATTAACTGTTAT
CCTACCATACTCTTGTAGTCAGTTCCTGAGATTACAGTGCCAGTCTGGAAAAGAAGAATCCAAACCCTCAGCTGTAGCAATATATATGGCTCATTTCTTTCCTTACT
CATTCTCAGCCAAGCAAATGCATTTTATTATCTTTAGTCACTGTCATCAAGACAGACACAATTGTCTGAAGATGTCACTAATTCTGGGTCGTTAAAGATGTCATTGT
TATAAGTTGTACCTGTGATTCAAATCCTTTCTTTTCTCTAGATACCTGTAATAACAGAGTCAGCAGGTGTGTGTGTGTGTGTCTGGGTGTGTGTGCGTGTGTGTG
TGTCTGGGTGTGTGTGTGTCTGTGTGTCTGTGTGTCAGTGTCTGTGTGTCTGTGTGTTTCTTAAGCTATGCCATTGTTCCTCTTAGTGTCTTTGTACATACTAGTAG
TTCAGAAAACAGTTTTCTAATTGTGGCTCATGGTGAGAAGATAGCTATTATAATTAGACAAACACTTTTTCTCTGAGCTTCTGATTTTTGCACTTATGAAAAGGGGA
TATAAAACAACTGGTTTCTGTGGTACCTTCCTATCCATCACGCTACATTCCACTAATACTCTACCTAGCTTCTATAGGGTTTGCTGTTATAACTAATGTCCAAGAGA
GCTCCCCTGTCAATATGATAGGCCTAAGTTAGGGGCAAAGTGAATACTGTTTTAAGAATCTTTAGTTGGGAGATGAAATAAGAATACTTGATGCAGCATAGTAGCCA
AAAGACTAAATTTAGGGATTGAATGCATAATATTACATCAGGTTTTAGAATGTGAAACCTCTACTGTCAAAAACCAAAAGTCAGCAAGGAGCTAGGGAAATGGTTCT
GAGGATAATGTGCTTGGTATACAAGCATGAGGACGTGAGCTTGGGTATCCTGCACTTACAGGAAGCTGGTCACAGAAGCATGCCTCTCTAAACTGGCTATGGGGCTT
GTGAGAGATGGAGACAAGAGAATTCTAAGGGCTTACTGGCCAACCAGTCTAAGCAAAATAGATTC
```

Rat
Chr7:92991347-92993522

```
GTTTGTTTGTTTGTTTGTTTGTTCGTTTGTTCACACACATGAAAATACTTTGAATCAAGAGATTGTGATCTAGGCCACACGGGGCCCTGAAAATCTGTTTGCTCAAG
TTCTAAGAACCTTCTGGAATTTAATAAAGACAGCATTCTATGTATGTGTGTTTAGGGCTGTATTCCTCTTCTGACAAAAGACCCTGTAGCATTTATTGTTAAGAAGT
TCATCACTGTACACAAGGGTTAAGAGCCATTGGCTTGAAATGGGGTTAGGAACATAAATTATTTATAGAGAAGCAATAATATTAACGTATATATTACAAGTCTCTTA
CTATTTGTGACTTGCCCATAATTCACCTCATTTGAGCTCAAGCTAAGCAGCTACACTAGTCAGAGCACAGCCCAGTGTAGTAACTAACACTGTATTCCTGGCCCTTA
CTATTAGAGGGCATTGATGTTTACCACCTTGACTTCAACTGCACAAGGCAAGGGCAAGGAGCACAGTATGACCTTAAACTATTCAACTCCACTTAATTACCCATTGT
AGTAGAAGCACATAAATACAGGATGTCCCTTTGCTCCCCCATTCTGCTTTGCATCATTATCAGAAAGACAAATGTAATTCATGTCCAATTACTCACTCACCGCAAGG
TCAGAATATTTATTTTTATCGTACTGGTGATTTTTAGGATTAGGGATATCAGTATTTATTCATGGCTAACCCAAACCAAACATCCTGTTGATATCTATGAATTGCTA
TATAGTTATTGTCACAGGTATGCTGAGTCACAGAGCAAATAAGAAGTTTATGAGTTGACAGGGAAAAGGCTGCAAGAGGGAAAGATAAGGTCCCCTGGGTAATCACT
GGTGTCTTGCGAAATAGAGGCATTGTTTACATTGTTCATCTATTGAGGACTGGGTGCTCGAGAAACTGAAACTAGCCCAAATAGTGAGGATTAAACAAAATTCACAT
CATTACTTTTAAAATGTGATGCTGGGACATTTTGATTTAATTTATGAACTGCTCATTTTAGCAATCAGTTTTTTTTTCCTCTGCCTCGTAGAGAAGAGCTTTGTCTA
GTTCTCAGATGTAGACAGAAGTAAGAGAGGTAGAAAACTAAAGGAAACTCTTCTGGGTAGAGTTCATTTGCTCAGAGATCTGGAGTAGAATTAACTGACGTCCTACC
GGGACCTTGGCAGTCAGTTCCTGAGATGAGAGTGACAGTCTTAAAAAGGAGAAGCCAAGCCCTCAACTGTATCAATGTGTATGGTTCATTTCTTTCCTTATTCACAT
TCAGCCAAGCAAATACACTTTATCTTCCGTCATTGTCATCAAGACAGAACTAATTGCCTGAAGATTTCACTAATTCTGGGTCTTTACAGATGGCCTTGTTATAAGTT
GTGCCTGAGATTCAAATCATTTCCTTTCTCTAGATACCTGTTATAACAGTTAACAGGTGTGTGAGTGTGTATGTGTGTGTGTCTGTCTGTGTGTCTGTGTGTCTGTG
TCTGTGTGTCTGTGTGTGGCTGTGTGTTTCTTAAGATGTGTCCTTATTCCTCCTCATATTTTTGTACATACTAGTAGTCTGGAGAACAGGCTTCTAATTGTGGTTCC
TAGGGAGAAGGTAGCTATTATAATTGGAAAAATACTTTTCCTCTGTGCTTCTGATTTTGCACTTATAAAAAGAGGACATAAAACAACTGGTGTCTGTGGTTCTATCC
TATCCATGATTCTATATTTAATTAGTACTCTATTTAGCTTCTATAGGGTATGCTGTCATAACTAATGTCCAAGACAGCTCCCCTGCCAATATGATAAGCCTAAATTA
GGGGTAATGTGAACAAAGTTTTAAGACTCTTTAGTGGAGAGAAGATGAAATTATAATCCTTGATGCAATGTAGTTTCCGAAGGACTAAAGTTGGGGTTTGAATGCAT
AATATTACATCAGTTTATAGAAATGTGAAGCCTTTATTCTCATGAATCAAACGTCATTGTGGAGCTAGCAAAATAGTTCTGAGGATAATGTACTTGATACACAAGGA
TGAGGACATGAGATTGGATACCCTGCACCCACAGGAAGCTGGTCATAGAAGCATGCATCTCTAAACTGGCTAAGGGGCTTGGGAGAGATGGAGAGAAGAGGCTTCAA
GGGCTTACTGGCCAGCCAGTCTAAGCAAAACAGGTC
```

Legend: **TCF sites** **SOX sites**

Fig S5. Sequences of the human Myc-521 WRE and homologues in mice and rats.
Positions shown are based on the GRCh38.p13 (human), GRCm39 (mouse) and mRatBN7.2 (rat) genome assemblies. Putative TCF and SOX9 binding sites are annotated and sites conserved in at least 2 species are underlined.

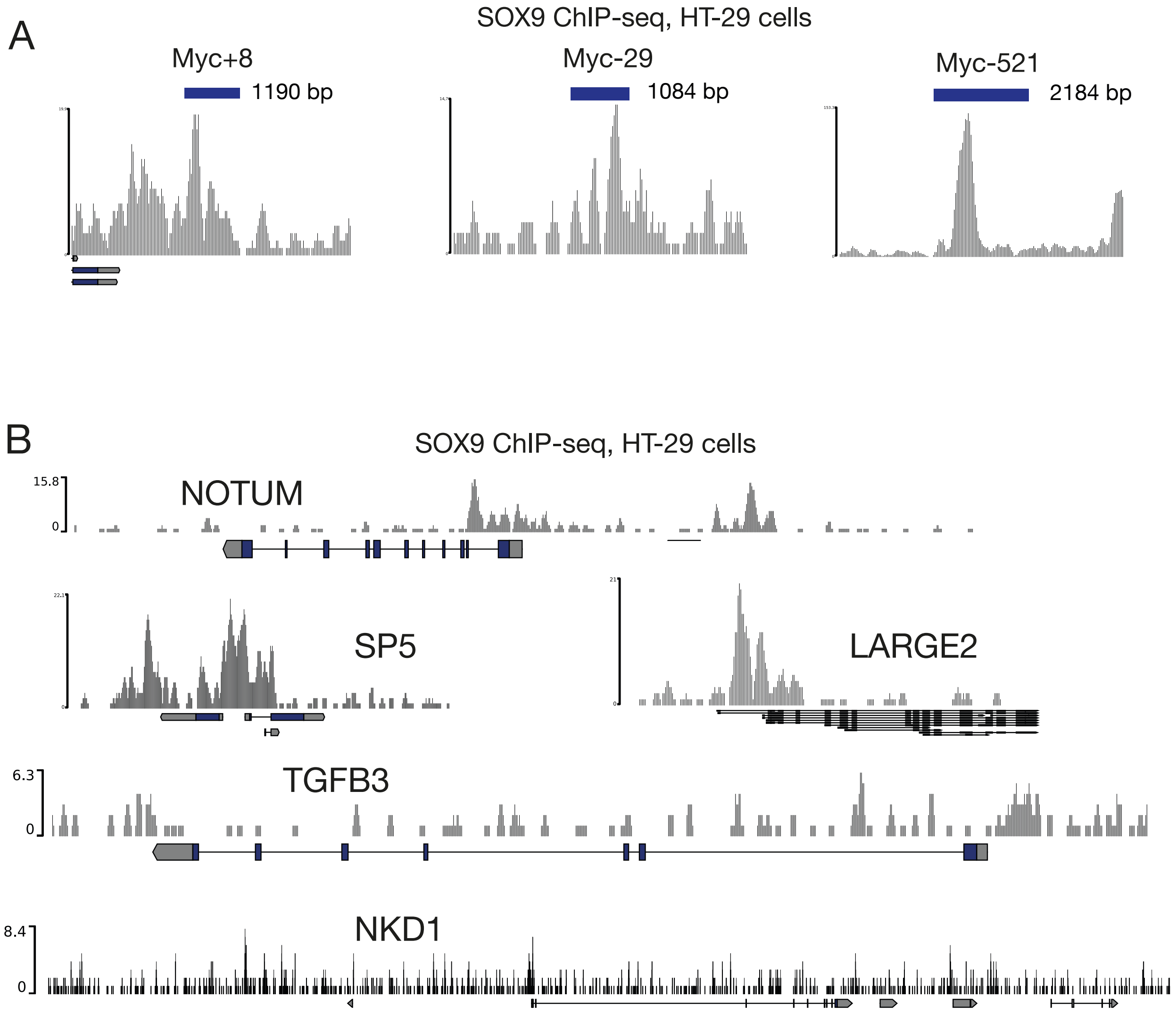

Fig S6. ChIP-seq tracks of SOX9-bound Wnt targets in CRC cells.
A, ChIP-seq tracks showing the binding of SOX9 to the Myc+8, Myc-29, and Myc-521 WREs. *B,* SOX9 ChIP-seq tracks of top 5 Wnt target genes in HT29 cells. SOX9 ChIP-seq dataset generated by Shi et al 2015.

A

Defa5 promoter sequence:
Chr8:7057354-7056678

GATGCATTCAGATCACACCAACTCCTTGAACTAAATCCGAATTTTTATTTTAATCTGATAAACTTGGCCTA
CTATTTTACTGAACTCATTTCCCCTATAGCCTGATAAGGTCATTGACCTCTCCATACTGGCACCAGCGGGA
GACTACTCACCTCGAGATCTCAAAAGCCTCCTACATGAGGTTAGTAATATCCCTGAATCCTGCAATGAATT
AACTCTCTACTCCACTGGGTCCCAGGTCTGCCCCCAGAGAGTCATCCAGAGAGTACCAGGGACCATCTTCA
GAAAACAAGAGGCATTTGATCCCCAAACTTCTTGAATGAAAGCGCTGTTGTTTTTCTTTTTTGAATATATA
AAAGTAAATACTCAAGCAGATGGGAAACAGAACAGGATAGTAATACCCTTATCATCATTAACACCTTGGAT
CAAGAAGAGGCATTAAGCATACAGACTCACGCTTTGATGAAAGCTGGGAGAAAGAGGAGCATCAAAGGGAT
CTTGAGAACAAAGGCAGTCCTTCCCCTCCCAATCACATGCCCACCTCCTCTCACTGCAGCTTCTGTCTCAG
GTCTTCTCCCAGCAGAGCTATAAATCCAGGCTGACTCCTCACTCCCCAC**ATATCCACTCCTGCTCTCCCTC**
CTGCAGGTGACCCCAGCCAGAGGACCATCGCCATCCT

Legend: TCF sites CDX sites CAG sites SOX sites **Exon**

B

Defa6 promoter sequence:
Chr8:6926680–6926025

TCACATGGGTTTCTTGAAATAAATCTTTTGGCTTTAGTTTTACTAACTCTTTACCTAGTATCCCACTGAGT
TCTTTTCCCTTATAGGCTGATAAGGTCATTATCTTCTCCACACTGTGCCCCACAAGAGCCTATTCACCCCA
TAATCTCAAAGCCTCCTTCATGAGGGCAGAAATGCCCCCTGAATCCTGCAATGAATTAACTCTCTACTCTA
GCGGGATCCAGCTCTGGCCTCAAGGTCTAGACCTCCAGAGAGTGGCCAGCCCCACCTTCAGAAAATAAGAG
GCATTTGATTCCTGAAATTATTCATTGAAAGCACTGTTCTTTTCTTTTTTGAATATTAACAAGTAAATATT
CCAGCAGATGGAAAACAGGACAATGTAACACTGTTCTTATCATCACTATCAGCTGGGACCAGAACAGACAC
TCAATAAACAGCCTCACACTACAATGAAGCTTGGAGAACAAAGGAGCATCAAAGGGACATGGAGGGCAAGG
GTAGCTCTTCTGCTCCCCAATCACATGCACTCCCCGTCTCACCGCAACATCTGTCCCTGAGCCTTCTCCCA
GCAGACCTATAAATCCAGGCTGGCTCCTCACTCCCC**ACACATCTGCTCCTGCTCTCTCTCCTCCAGCGACC**
CTAGCCAGtAGAACCCT

C

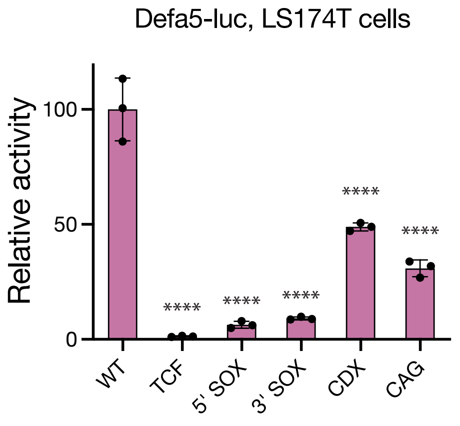

D

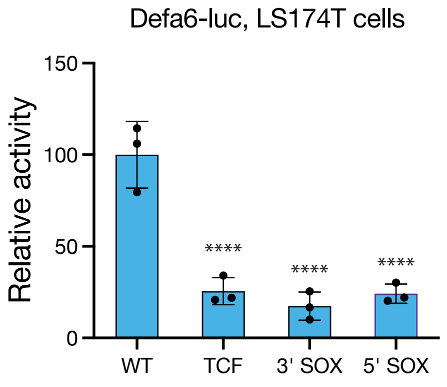

Fig S7. Synergistic upregulation of Paneth cell defensins by Wnt signalling and SOX9.
*A***,** sequence of the *Defa5* WRE with regulatory motifs annotated. The region between nucleotides -617 to +60 with respect to the transcription start site (TSS) were cloned to make the reporter. The ATG at +40 was changed to AGT in the reporter. *B,* sequence of the *Defa6* WRE with regulatory motifs annotated. Positions shown are based on the GRCh38.p13 primary assembly of the *Homo sapiens* genome. The region between nucleotides -604 to +52 with respect to the TSS were cloned into the promoter. ATG at +41 was changed to AGT in the reporter. *C,* luciferase assay data showing a reduction in Defa5-luc activity in LS174T cells upon the mutation of TCF, SOX, CDX, or CAG sites. *D,* luciferase assay showing a reduction in Defa6-luc activity in LS174T cells upon mutation of TCF or SOX sites. Data in (C) and (D) are shown as mean ± s.d. of biological triplicates. ns $P > 0.05$, *$P < 0.05$,** $P < 0.01$, *** $P < 0.001$. **** $P < 0.0001$. P-values were calculated using one-way ANOVA followed by Dunnett's test.

A

TOPflash
GGTACCTGAGCTCCACCGCGGTGGCGGCCGCTCTAGAACTAGTGGATCCCCCGG**GAGATCAAAGG**GGG
T**AAGATCAAAGG**GGGT**AAGATCAAAGG**GGTCGG**GAGATCAAAGG**GGGT**AAGATCAAAGG**GGGT**AAGAT**
CAAAGGGGTCGACCTCGAGGATATCAAGATCTGGCCTCGGCGGCCAAGCTT

B

SOXflash
AAATAGGCTGTCCCCAGTGCAAGTGCAGGTGCCAGAACATTTCTCTGGCCTAACTGGCCGGTACCTGA
GCTCGCTAGCCTCGAGATTT**AACAATGT**GT**TCTTTGTC**CCACATTT**AACAATGT**GT**TCTTTGTC**CCAC
ATTT**AACAATGT**GT**TCTTTGTC**CCACAGATCTGGCCTCGGCGGCCAAGCTT

C

TOP/SOX
ACGAAACAAAACAAACTAGCAAAATAGGCTGTCCCCAGTGCAAGTGCAGGTGCCAGAACATTTCTCTG
GCCTAACTGGCCGGTACCTGAGCTCGCTAGCCTCGAGTCGG**AACAATGT**G**TTCTTTGT**GTAC**GAGATC**
AAAGGGGGT**AACAATGT**G**TTCTTTGT**GTAC**AAGATCAAAGG**GGTCAAGCTT

Legend: **TCF sites** **SOX sites**

Fig S8. Sequence determinants of synergistic activation by Wnt signalling and SOX9.
Sequences of the (A) TOPflash, (B) SOXflash, and (C) TOP/SOX synthetic reporters with TCF and SOX sites annotated.

A

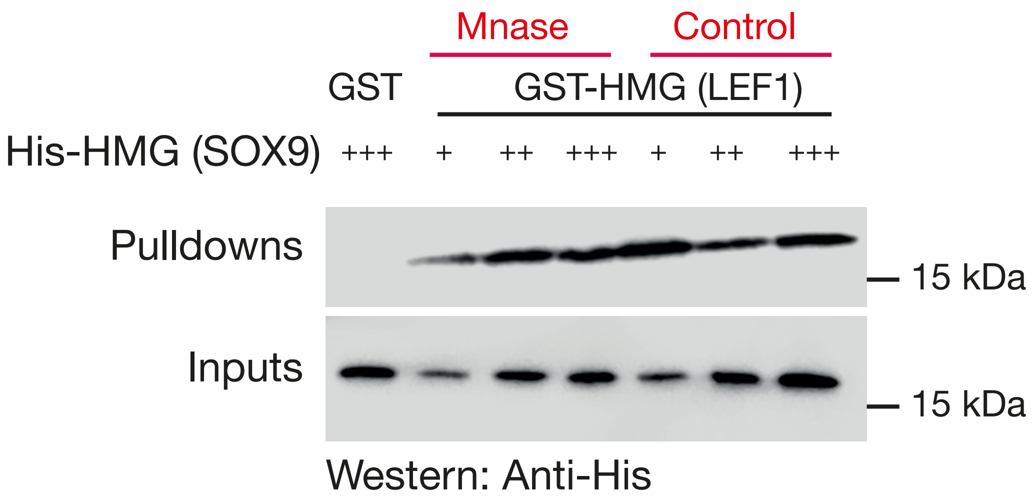

B

Uniprot ID, species, protein	Sequence	Positions
Q04887<br>Mouse SOX9	KLADQYPHLHNAELSKTLGKLWRLLNESEKRPFVEEAERLRVQHKKDHPDY	122-172
P48436<br>Human SOX9	KLADQYPHLHNAELSKTLGKLWRLLNESEKRPFVEEAERLRVQHKKDHPDY	122-172
A0A337SCT0<br>Cat SOX9	KLADQYPHLHNAELSKTLGKLWRLLNESEKRPFVEEAERLRVQHKKDHPDY	122-172
P48434<br>Chick SOX9	KLADQYPHLHNAELSKTLGKLWRLLNESEKRPFVEEAERLRVQHKKDHPDY	122-172
Q6F2E7<br>X. tropicalis SOX9	KLADQYPHLHNAELSKTLGKLWRLLNEGEKRPFVEEAERLRIQHKKDHPDY	122-172
Q9DFH1<br>D. rerio SOX9b	KLADQYPHLHNAELSKTLGKLWRLLNEGEKRPFVEEAERLRVQHKKDHPDY	108-158
Q9DFH2<br>D. rerio SOX9a	KLADQYPHLHNAELSKTLGKLWRLLNEVEKRPFVEEAERLRVQHKKDHPDY	124-174
Q9VA17<br>D. melanogaster SOX100B	VMSKQYPHLQNSELSKSLGKLWKNLKDSDKKPFMEFAEKLRMTHKQEHPDY	94-144
Q23045<br>C. elegans egl-13	KILKAYPDMHNSNISKILGSRWKGMSNSEKQPYYEEQSRLSKLHMEQHPDY	346-396

Fig S9. Highly conserved residues mediate a DNA-independent TCF/SOX9 interaction.
A, western blot analysis of a GST pulldown assay showing that the interaction of the HMG domain of LEF1 (GST-tagged) with the HMG domain of SOX9 (His tagged) is unaffected by treatment with Mnase, suggesting that it does not require DNA. *B,* sequence comparison of SOX9 proteins across species showing conservation of residues mediating the TCF/SOX9 interaction.

Protein	HMG sequence	Positions
SOX9	RRKLADQYPHLHNAELSKTLGKLWRLLNESEKRPFVEEAERLRVQHKKDHPDYK	120-173
SOX8	RRKLADQYPHLHNAELSKTLGKLWRLLSESEKRPFVEEAERLRVQHKKDHPDYK	117-170
SOX10	RRKLADQYPHLHNAELSKTLGKLWRLLNESDKRPFIEEAERLRMQHKKDHPDYK	119-172
SOX7	RKRLAVQNPDLHNAELSKMLGKSWKALTLSQKRPYVDEAERLRLQHMQDYPNYK	60-113
SOX17	RKRLAQQNPDLHNAELSKMLGKSWKALTLAEKRPFVEEAERLRVQHMQDHPNYK	83-136
SOX18	RKRLAQQNPDLHNAVLSKMLGKAWKELNAAEKRPFVEEAERLRVQHLRDHPNYK	100-153
SOX4	RRKIMEQSPDMHNAEISKRLGKRWKLLKDSDKIPFIREAERLRLKHMADYPDYK	74-127
SOX11	RRKIMEQSPDMHNAEISKRLGKRWKMLKDSEKIPFIREAERLRLKHMADYPDYK	64-117
SOX12	RRKIMDQWPDMHNAEISKRLGRRWQLLQDSEKIPFVREAERLRLKHMADYPDYK	55-108
SOX5	RRKILQAFPDMHNSNISKILGSRWKAMTNLEKQPYYEEQARLSKQHLEKYPDYK	571-624
SOX6	RRKILQAFPDMHNSNISKILGSRWKSMSNQEKQPYYEEQARLSKIHLEKYPNYK	636-689
SOX13	RRKILQAFPDMHNSSISKILGSRWKSMTNQEKQPYYEEQARLSRQHLEKYPDYK	439-492
SOX1	RRKMAQENPKMHNSEISKRLGAEWKVMSEAEKRPFIDEAKRLRALHMKEHPDYK	66-119
SOX2	RRKMAQENPKMHNSEISKRLGAEWKLLSETEKRPFIDEAKRLRALHMKEHPDYK	56-109
SOX3	RRKMALENPKMHNSEISKRLGADWKLLTDAEKRPFIDEAKRLRAVHMKEYPDYK	154-207
SOX14	RRKMAQENPKMHNSEISKRLGAEWKLLSEAEKRPYIDEAKRLRAQHMKEHPDYK	23-76
SOX21	RRKMAQENPKMHNSEISKRLGAEWKLLTESEKRPFIDEAKRLRAMHMKEHPDYK	23-76
SOX15	RRQMAQQNPKMHNSEISKRLGAQWKLLDEDEKRPFVEEAKRLRARHLRDYPDYK	64-117
SRY	RRKMALENPRMRNSEISKQLGYQWKMLTEAEKWPFFQEAQKLQAMHREKYPNYK	75-128
SOX30	RPALAKANPAANNAEISVQLGLEWNKLSEEQKKPYYDEAQKIKEKHREEFPGWV	352-405

Fig S10. Conservation of TCF-interacting residues across human SOX9 proteins.
When mutated in SOX9, D125,168,171 prevent it from binding to the HMG domain of TCFs. Amino acid residues in other human SOX family members that have acidic (D/E) residues at the corresponding positions are highlighted.

A

	D125	D168	D171
SOX9	D	D	D
SOX2	Q	E	D
SRY	L	K	N
SOX7	V	D	N

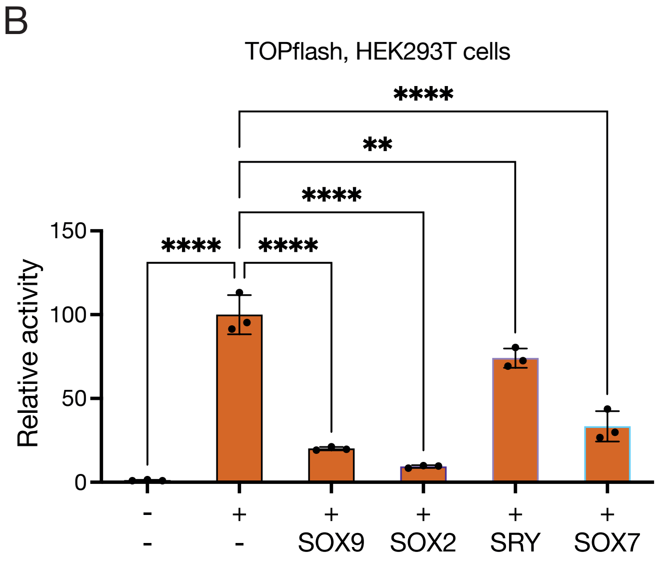

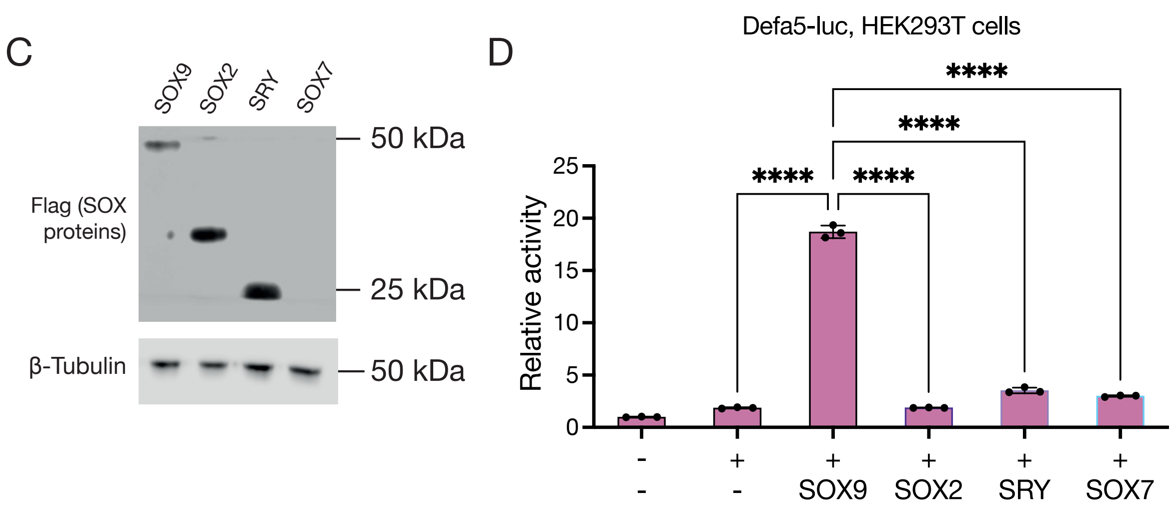

Fig S11. SOX9 is unique in its ability to activate the *Defa5* promoter.
A, Table showing the identity of amino acid residues in human SOX2, SRY, and SOX7 proteins at positions corresponding to human SOX9 residues D125, D168, and D171. *B,* Luciferase assay showing repression of TOPflash activity by SOX9, SOX2, SRY, and SOX7. *C,* western blots against the Flag tag (to detect Flag-tagged SOX proteins) and β-Tubulin in HEK293T cells transfected for the experiment in (A). Although SOX7 potently suppressed TOPflash activity, it could not be detected on a western. *D,* Luciferase assay showing only SOX9 cooperates with β-catenin to activate the *Defa5-luc* reporter in HEK293T cells. For (B) and (D), HEK293T cells were transfected with indicated reporters and plasmids expressing β-catenin* and indicated SOX proteins. Luciferase activity was measured 24h later. Bars represent mean ± s.d. of biological triplicates. ns P > 0.05, *P < 0.05 ,** P < 0.01, *** P < 0.001. **** P < 0.0001. P-values were calculated using one-way ANOVA followed by Dunnett's test.

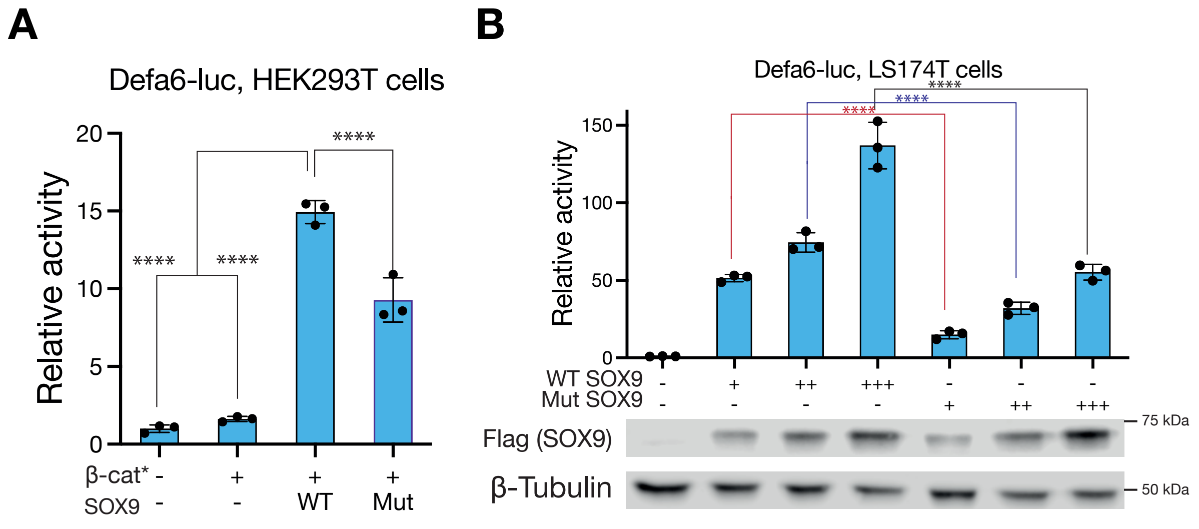

Fig S12. Non-DNA contacting TCF-interacting residues play a role in the activation of Wnt-SOX9 activated enhancers.

Luciferase assay data showing the activation of Defa6-luc by β-catenin* and WT or Mut SOX9 in HEK293T cells (A) and by WT or Mut SOX9 in LS174T cells (B). Western blots in (B) show relative expression levels of WT and Mut SOX9 with Tubulin as loading control. Each bar represents mean ± SD from three replicates (N = 3). ns $P > 0.05$, *$P < 0.05$,** $P < 0.01$, *** $P < 0.001$. **** $P < 0.0001$. P-values were calculated using one-way ANOVA followed by Dunnett's test.

Construct	Target gene	pGIPZ clone ID	Target sequence
Scr	Scrambled negative control	RHS4346	ATCTCGCTTGGGCGAGAGTAAG
SOX9-1	SOX9	V2LHS_92504	TAAGACTGCAGTGAACAAG
SOX9-2	SOX9	V2LHS_11387	ATGTCTTGAAGGTTAACTG

Table S1. Targeting sequences and clone IDs of the pGPIZ RNAi clones used to create LS174T cell lines expressing scrambled (Scr) or SOX9-targeting shRNAs.
"Target sequence" denotes the sequence of the mature antisense siRNA.

Construct	Target sequence
SOX9 RNAi / shRNA 1	AAGCTCTGGAGACTTCTGA
shRNA 2	ACCACCCGGATTACAAG
shRNA 3	TTGTTCACTGCAGTCTTAA

Table S2. Targeting sequences of SOX9 targeting shRNA sequences cloned into pSUPER for transient transfection RNAi experiments

Construct	Site	Original site	Mutated site
5' SOX	SOX9	AACAATGT	AAaccTGT
3' SOX	SOX9	TCTTTGTC	gaTgTGTC
TCF+SOX	SOX9 (3' SOX)	TCTTTGTC	gaTgTGTC
	TCF	CCTTTGAGC	CCTgTtcGC
		GATGAAAGG	GAgtAcAGG
		GTACAAAGA	GTACAcAtc
		AATCAAAGG	AAgaAcAGG

Table S3. Site-directed mutagenesis of the c-Myc-335 reporter

Reporter	Site	Original	Mutated
**Defa5**	**TCF**	GCTTTGATG	GCTGTGCGG
		CATCAAAGG	CCGCACAGG
		GAACAAAGG	GCCCACAGG
	**5' SOX**	ATGAAAGC	ATTCCAGC
	**3' SOX**	CTGTTGTT	CTGCGTTT
	**CAG**	TACTGA	TTCACA
		TACTGG	TTCACG
		CACTGG	CTCACG
		ACAGAC	AGACTC
		GGCTGA	GCCACA
		CCAGCC	CGTGGC
**Defa6**	**TCF**	TCTCAAAGC	TCGTACAGC
		ATTGAAAGC	AGGGACAGC
		GAACAAAGG	GCCCACAGG
		CATCAAAGG	CCGCACAGG
	**5' SOX**	CTATTCAC	CGATGCCC
		TCCTTCAT	TACTGCCT
		GCTTGGAG	GATTTGCG
		ACATGGAG	AAATTGCG
	**3' SOX**	CACAAGAG	CAACCGAG
		CTCAAAGC	CTACCAGC
		TACAATGA	TAACCTGA

Table S4. Site-directed mutagenesis of the *Defa5/6* reporters

Target	Primer
**Defa5**	ACCTCAGGTTCTCAGGCAAG
	CTGATTTCACACACCCCGGA
**G6PD (control)**	GAGGCCGTGTACACCAAGAT
	TCAGGGAGCTTCACGTTCTT
**Myc**	TACAACACCCGAGCAAGGAC
	AGCTAACGTTGAGGGGCATC

Table S5. Primers used for RT-qPCR

Target	Primer
**c-Myc-335 (Peak)**	TCCTATCTCAGCTCCCTATCCA
	CTGCTGAGCTCAAAGGACGA
**c-Myc-335 flank 1**	GGCTCTCACCCTTCAACCAA
	GCTTGTGACTTAGCCTGGGT
**c-Myc-335 flank 2**	GGCTCTGGTTGGGGGTTTTA
	TGAGGCGGAAGTCAACACAG
**Control**	CGATTCAGTGCCGCATTAGC
	TAATCGGCTGGATCTCCCCA

Table S6. Primers used for ChIP-qPCR
